# Soybean glyceollins mitigate inducible nitric oxide synthase and cyclooxygenase-2 expression levels via suppression of the NF-κB signaling pathway in RAW 264.7 cells

**DOI:** 10.3892/ijmm.2012.887

**Published:** 2012-01-12

**Authors:** EUN-KYUNG YOON, HYUN-KYOUNG KIM, SONG CUI, YONG-HOON KIM, SANG-HAN LEE

**Affiliations:** 1 Department of Food Science and Biotechnology, Kyungpook National University, Daegu 702-701; 2 Food and Bio-Industry Research Institute, Kyungpook National University, Daegu 702-701; 3 N&B Co., Ltd., Techno Building, Kyungpook National University, Daegu 702-832, Republic of Korea

**Keywords:** glyceollins, NF-κB, inducible nitric oxide synthase, cyclooxygenase-2, anti-inflammation

## Abstract

Glyceollins, produced to induce disease resistance responses against specific species, such as an incompatible pathogen *Phytophthora sojae* in soybeans, have the potential to exhibit anti-inflammatory activity in RAW 264.7 cells. To investigate the anti-inflammatory effects of elicited glyceollins via a signaling pathway, we studied the glyceollin signaling pathway using several assays including RNA and protein expression levels. We found that soybean glyceollins significantly reduced LPS-induced nitric oxide (NO) and prostaglandin E2 (PGE_2_) production, as well as the expression of inducible NO synthase (iNOS) and cyclooxygenase-2 (COX-2) via the suppression of NF-κB activation. Glyceollins also inhibited the phosphorylation of IκBα kinase (IKK), the degradation of IκBα, and the formation of NF-κB-DNA binding complex in a dose-dependent manner. Furthermore, they inhibited pro-inflammatory cytokines, such as tumor necrosis factor (TNF)-α, interleukin (IL)-1β and IL-18, but increased the generation of the anti-inflammatory cytokine IL-10. Collectively, the present data show that glyceollins elicit potential anti-inflammatory effects by suppressing the NF-κB signaling pathway in RAW 264.7 cells.

## Introduction

**Figure 1:**
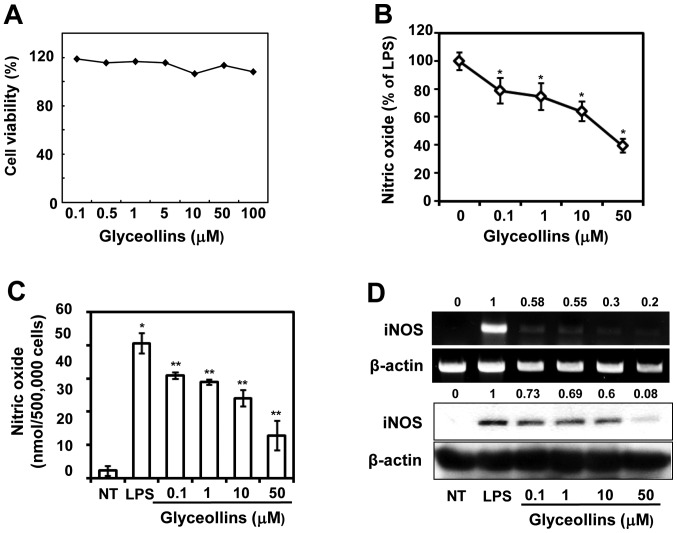
Effect of glyceollins on nitric oxide production and iNOS in LPS-treated RAW 264.7 cells. (A) RAW 264.7 cells were treated with the indicated dose of glyceollins for 48 h. Cell viability was determined using an MTT assay. (B and C) RAW 264.7 cells were pretreated for 2 h with indicated concentrations of glyceollins before LPS (1 μg/ml) stimulation for 16 h, and the amount of nitrite in the supernatant from each treatment group was measured using Griess reagent. Values are the means ± SD of three independent experiments. (B) ^*^P<0.05, compared to the untreated as control. (C) ^*^P<0.05, compared to the control; ^**^P<0.05, compared to the LPS treatment. (D) Nitric oxide synthase mRNA and protein expression were examined by measuring RT-PCR analysis with iNOS primers or western blotting using an anti-iNOS antibody.

It is now recognized that soybeans (*Glycine max*) and soybean foods, such as tofu and soymilk, can contribute to the better health of the elderly, young people, and pregnant woman (1). The soybean, a legume with a high protein content (~35–40%) is excellent at meeting dietary protein needs. Moreover, the importance of soybeans is proven by clinical and preclinical studies of hypocholesterolemia, diabetes, obesity, renal dysfunction and cardiovascular disorders (2,3). Soybeans contain many known components, such as phytoalexins, that are incredibly useful. Genistein, a soybean isoflavone, has been highlighted over the last few years as a potentially beneficial agent due to its wide-ranging effects on breast cancer, osteoporosis, coronary heart disease, diabetes and menopausal discomfort (4–6). Glyceollin, a phytoalexin found in soybeans and grapes, is produced as an immune response to pathogens (7). Glyceollins are isolated as a mixture of glyceollin I, II, and III (Fig. 1A), from germinated soybean from *Rhyzopus* sp. by an elicitor (8). These compounds have been investigated in relation to their anti-estrogenic activity through the inhibition of estrogen receptor α and β compared with well-known phytoestrogenic chemicals such as enterolactones and genistein (9–11). It also has been recognized that glyceollins can suppress human breast and ovarian carcinoma tumorigenesis (12) and may modulate potential estrogenic properties in the breast through an anti-estrogenic effect (13); however, researchers have yet to elucidate the molecular mechanisms of their biological activity.

On the other hand, it has been revealed that NF-κB plays a pivotal role in molecular inflammation by controlling the expression of various genes that encode pro-inflammatory cytokines, chemokines and inducible enzymes, such as inducible nitric oxide synthase (iNOS) and cyclooxygenase-2 (COX-2), in mammalian immune cells (14). NF-κB is ubiquitously located in the cytoplasm of non-stimulated cells because of interactions with inhibitory proteins such as IκBs (15); but, it responds to pro-inflammatory stimuli, when IκBs are rapidly phosphorylated and degraded by the 26S proteasome (16,17), which results in the dissociation of free NF-κB dimers of p65 and p50, which translocate to the nucleus, and then activate the transcription of target genes. The IκBα kinase (IKK) complex contains two catalytic subunits: i) IKKα/β and ii) a regulatory subunit, IKKγ (18–20). Activation of IKK is mediated by phosphorylation through various upstream kinases, such as the NF-κB-inducing kinase, NF-κB-activating kinase, Akt, and protein kinase Cζ, that are involved in cellular signaling in response to pro-inflammatory stimuli (21). Following activation, the NF-κB heterodimer activates the transcription of target genes, including the genes encoding the pro-inflammatory cytokines, such as interleukin (IL)-1, -6, -8, -18 and tumor necrosis factor-α (TNF-α) as well as iNOS, COX-2 and cell adhesion molecules (22–24). In turn, the products regulated by NF-κB, such as TNF-α and IL-1β, also lead to the activation of NF-κB. This fact means that there is a complex regulatory loop that amplifies and perpetuates inflammatory responses; therefore, it has become a biological target for new types of anti-inflammatory treatment.

Based on this reason, we first hypothesized that glyceollins, overproduced by an elicitor *Rhyzopus* sp., can ameliorate immune cells by an NF-κB signaling pathway. To prove this hypothesis, we compared whether glyceollins have the potential to possess potent anti-inflammatory effects by inhibiting LPS-induced iNOS and COX-2 expression, as well as various inflammatory cytokines like TNF-α, IL-1β, IL-18 in RAW 264.7 cells. We further examined whether glyceollins inhibit the LPS-induced inflammation process by blocking an NF-κB signaling pathway.

## Materials and methods

### Materials and cell culture

LPS (purified from *Escherichia coli* 055:B5) was purchased from Sigma-Aldrich (St. Louis, MO) and fetal bovine serum (FBS) was purchased from Invitrogen (Carlsbad, CA). Antibodies against iNOS, COX-2, IKKα/β, NF-κB p65, IκBα and phospho-IκBα (Ser-32/36) were obtained from Cell Signaling Technology (Beverly, MA). RAW 264.7 cells were cultured in a RPMI-1640 medium supplemented with 10% of FBS, 2 mM of L-glutamine, 100 U/ml of penicillin, and 100 μg/ml of streptomycin at 37˚C in a 5% CO_2_ humidified incubator. We measured cell viability by counting, with a stain of 0.2% trypan blue solution.

### Preparation of glyceollin mixture by elicitation

Glyceollins I, II and III (Fig. 1A, B and C) were semi-purified from elicited soybeans, with a slight modification, as previously described (8,11). In brief, we used soybeans (AGA, variant no. 3, a gift from KNU Soyventure, Daegu, Korea) that overproduce isoflavones to the rate of 10 mg/g of soybeans. The soybeans were elicited by a *Rhyzopus* sp. for *de novo* synthesis of glyceollins, which produced three kinds of isomers (data not shown). The cultures of *Rhyzopus* sp. were grown at 25˚C in a culture room on potato dextrose agar media and the inoculums were harvested after 5 days. We first washed the soybeans by dipping in a 65% ethanol solution for 1 min; thereafter, we washed them with deionized water before the soaking process. Soybean seeds were soaked in sterile, deionized water for 6 h, and then placed into a Sanyo MLR-351H growth chamber (Carlsbad, CA). After saturation with distilled water, the seeds were cut into 4 pieces by a knife. A spore suspension (100 μl) of *Rhyzopus* sp. was dispersed on the cut surfaces of the seeds. The seeds (20 g) were incubated at 26˚C for 4 days, extracted with 50 ml of 80% (v/v) ethanol for 1 h, then cooled and centrifuged at 20,000 × g for 10 min. The extracts were filtered by a membrane filter (0.45 μm) from Sartorius (Aubagne Cedex, France). Each crude extract was freeze-dried and dissolved in dimethyl sulfoxide. The extract was 100 μg/ml, and was used for further purification. For purification, we used a high-performance liquid chromatography system, the HPLC, PerkinElmer Series 200 (Waltham, MA). A Brownlee Choice C18 (150×4.6 mm) reverse-phase column and guard column were used. The injection volume and column temperature were 10 and 30 μl, respectively. Detection was monitored at 260 and 285 nm, and the flow rate was 1.0 ml/min at the following solvent condition (A, acetonitrile, B, 1% acetate in water; 0 to 45% A for 10.2 min; 45 to 90% A for 6 min, holding at 90% A for 3.6 min). The glyceollins were separated by thin-layer chromatography (TLC) for use in further experiments because the concentration was low and the amounts that we needed were small. The glyceollin phase was separated by open-column chromatography (OCC). A methanolic extract was fractionated on a Silica gel-60 (<0.063 mm: 0.063 mm, 8:2) developed in methylene chloride:methanol (97–99:1–3, v/v) solution. A sample of OCC extract was fractionated on aluminum-backed Silica gel-60 TLC plates developed in hexane:acetone (4:1.5, v/v). Glyceollins were visualized with 20% aqueous sulfuric acid spray reagent, and UV light at 254 nm. A single glyceollin band (Rf=0.2) was confirmed by a standard glyceollin, and further purified by preparative TLC. The concentration of glyceollin isomers in the fraction was up to ~90% as analyzed by preparative HPLC. The relative ratio of glyceollin I, II and III in the fraction was 17:2:1. Thereafter, we designated the fraction as glyceollins.

### Cell viability assay

Cell viability was measured by the MTT [3-(4,5-dimethylthiazol-2-yl)-2,5-diphenyltetrazolium bromide] assay (25). Cells were seeded on 96-well plates at a concentration of 5×10^4^ cells/well. After seeding, cells were incubated with MTT solution (1 mg/ml) for 2 h. The medium was discarded and the formazan product was solubilized with dimethyl sulfoxide. Viability was assessed by measuring absorbance at 570 nm with a Bio-Rad microplate reader (Hercules, CA).

### Nitrite quantification

RAW 264.7 cells were treated with 1 μg/ml LPS plus sample for 16 h. Nitric oxide synthesis can be determined by assaying nitrite in the culture supernatant because nitrite is a stable reaction product of NO (26). Briefly, cell-free culture media were reacted with a 1:1 mixture of Griess reagent (1% sulfanilamide, 0.1% naphthylethylenediamine dihydrochloride and 2.5% phosphoric acid) at room temperature for 10 min. The OD of the assay sample was measured spectrophotometrically at a wavelength of 570 nm. Nitrite concentration was calculated from a standard curve prepared using NaNO_2_ under the same assay conditions.

### Western immunoblot analysis

Proteins were separated by SDS-PAGE and immunoblotted onto a nitrocellulose membrane in a buffer containing 20% methanol, 25 mM of Tris, and 192 mM of glycine, as described elsewhere (27). The membranes were then blocked with 5% non-fat dry milk and incubated with the primary antibody overnight. Subsequently, membranes were washed in Tween-Tris buffer saline (TTBS), incubated for 4 h with horseradish peroxidase-conjugated goat anti-mouse or goat anti-rabbit (1:4,000) antibodies, and finally developed using an enhanced ECL system (KPL Inc., Gaithersburg, MD). The membranes were then reprobed with a β-actin antibody as a control.

### Reverse transcription-polymerase chain reaction

Total-RNA was extracted from RAW 264.7 cells using an easy-BLUE total-RNA extraction kit from Intron Biotechnology (Sungnam, Korea) according to the manufacturer's protocols and was quantified by measuring absorbance at 260 nm. RNA was reverse-transcribed using 2.5 μM oligo(dt) primers, 1 mM dNTPs, and Moloney murine leukemia virus (MMLV) reverse transcriptase (Promega, Madison, WI), and the resulting cDNAs were amplified with SuperTherm DNA polymerase SR Product (Kent, UK). β-actin primers were used to standardize the amount of RNA in each sample. PCR products were resolved on 1% agarose gels and visualized by ethidium bromide staining (28).

### Measurement of prostaglandin E2 production

RAW 264.7 cells were subcultured in 6-well plates treated with the indicated dose of glyceollins for 2 h in the presence or absence of LPS (1 μg/ml) for 16 h (29). The culture media was collected for determination of prostaglandin E2 (PGE_2_) concentration by an enzyme immunoassay kit from Cayman (Ann Arbor, MI).

### Nuclear extracts

RAW 264.7 cells were incubated with glyceollins and LPS as indicated (30). The cells were harvested in PBS containing 2% serum, washed twice with ice-cold PBS, and resuspended in 500 μl of buffer A (10 mM of HEPES, pH 7.9, 5 mM of MgCl_2_, 10 mM of KCl, 1 mM of ZnCl_2_, 0.2 mM of EGTA, 1 mM of Na_3_VO_4_, 10 mM of NaF, 0.5 mM of dithiothreitol, 0.5 mM of PMSF and protease inhibitors). After the cells had been incubated on ice for 10 min and lysed by adding 50 μl of 20% Nonidet P-40, to a final concentration of 2%, their nuclei were harvested by centrifugation. The nuclear pellets were then resuspended in 60 μl of extraction buffer (10 mM HEPES, pH 7.9, 5 mM MgCl_2_, 300 mM NaCl, 0.2 mM EGTA, 25% glycerol, 1 mM Na_3_VO_4_, 10 mM NaF, 0.5 mM dithiothreitol, 0.5 mM PMSF and protease inhibitors), and incubated on ice for 15 min. Nuclear debris was then removed by centrifugation (13,000 rpm, 10 min), and the nuclear extracts were subjected to gel shift analysis. Protein concentrations were determined using a Bradford method (31).

### Electrophoretic mobility shift assay

RAW 264.7 cells, in 10-cm diameter dishes (10^7^ cells/dish), were pretreated with or without glyceollins for 2 h and then incubated with LPS (1 μg/ml) for 16 h. For the gel shift assay, a consensus sequence for the NF-κB DNA binding site, sc-2505 from Santa Cruz Biotechnology, Inc. (Santa Cruz, CA), was used. The mutant binding sequence for NF-κB was identical to sc-2505 except for a G→C substitution in the NF-κB-DNA binding motif (sc-2511, Santa Cruz Biotechnology, Inc.). Beforehand, the NF-κB probe was biotin-end labeled by the biotin 3′ end DNA labeling kit from Thermo Scientific Pierce (Rockford, IL). Briefly, EMSA binding reactions were performed by incubating 20 μg of nuclear extract with the annealed oligos with the LightShift EMSA kit from Thermo Scientific Pierce, according to the manufacturer's instructions. The reaction mixture was subjected to electrophoresis on a 4% native gel in a 0.5xTBE buffer. After transfer, the membrane was immediately cross-linked for 15 min on a UV transilluminator equipped with 312 nm bulbs. A chemiluminescence detection method utilizing a luminol/enhancer solution and a stable peroxide solution from Thermo Scientific Pierce was used as described by the manufacturer's manual, and the membranes were exposed to X-ray films for 2–5 min before developing (32).

### Statistical analysis

Statistical differences between mean values ± SD were determined by the Dunnett's multiple range test. The significance was set at P<0.05 (33).

## Results

### Effect of glyceollins on cell viability

To examine whether glyceollins exhibit cytotoxicity in cells, we first measured whether glyceollins affect cell proliferation in RAW 264.7 cells. The result showed that, at concentrations up to 100 μM, glyceollins had no toxic effect on cell viability (Fig. 1A). Major and/or minor fractions did not have any morphological changes in microscopic observation (data not shown). Therefore, we decided to investigate whether glyceollins have potential in reducing iNOS and COX-2 expressions, which could be a landmark for the assessment of molecular inflammation.

### Effects of glyceollins on NO production and expression of iNOS in LPS-stimulated RAW 264.7 cells

To assess the inhibitory effect of glyceollins on LPS-induced NO production in RAW 264.7 cells, the cells were treated with LPS (1 μg/ml) for 16 h after treatment in the presence or absence of glyceollins (0.1, 1, 10 or 50 μM) for 2 h. The amount of nitrite, a stable metabolite of NO, was used as the indicator of NO production in the medium. During the 16 h of incubation, RAW 264.7 cells produced up to 6.4±0.02 μM of nitrite in the resting state. When LPS (1 μg/ml) was added, NO production was dramatically increased up to 56.2±0.01 μM (Fig. 1B). In this condition, adding glyceollins inhibited LPS-induced NO production in a concentration-dependent manner corresponding to 10.6 and 58.7% inhibition at 0.1 and 50 μM, respectively (Fig. 1C). To further investigate whether the inhibitory effect of glyceollins on NO production was associated with the inhibition of corresponding gene expression, the protein and mRNA expressions of iNOS were determined by semi-quantitative RT-PCR and western blot analysis, respectively. In unstimulated RAW 264.7 cells, the iNOS mRNA and protein expressions were almost undetectable (Fig. 1D, first bands of each set); however, LPS treatment augmented the protein and mRNA expressions of iNOS remarkably; pretreatment of the cells with different concentrations of glyceollins also dramatically reduced LPS-induced iNOS mRNA and protein expressions in a concentration-dependent fashion (Fig. 1D, compare the second and sixth bands of each set at 0 to 50 μM). The intensity was a 5- and 12.5-fold decreased compared with that of LPS-treated cells. The data suggest that glyceollins can downregulate LPS-induced iNOS expression at the transcription level.

### Effects of glyceollins on PGE_2_ production and COX-2 expression in LPS-stimulated RAW 264.7 cells

**Figure 2:**
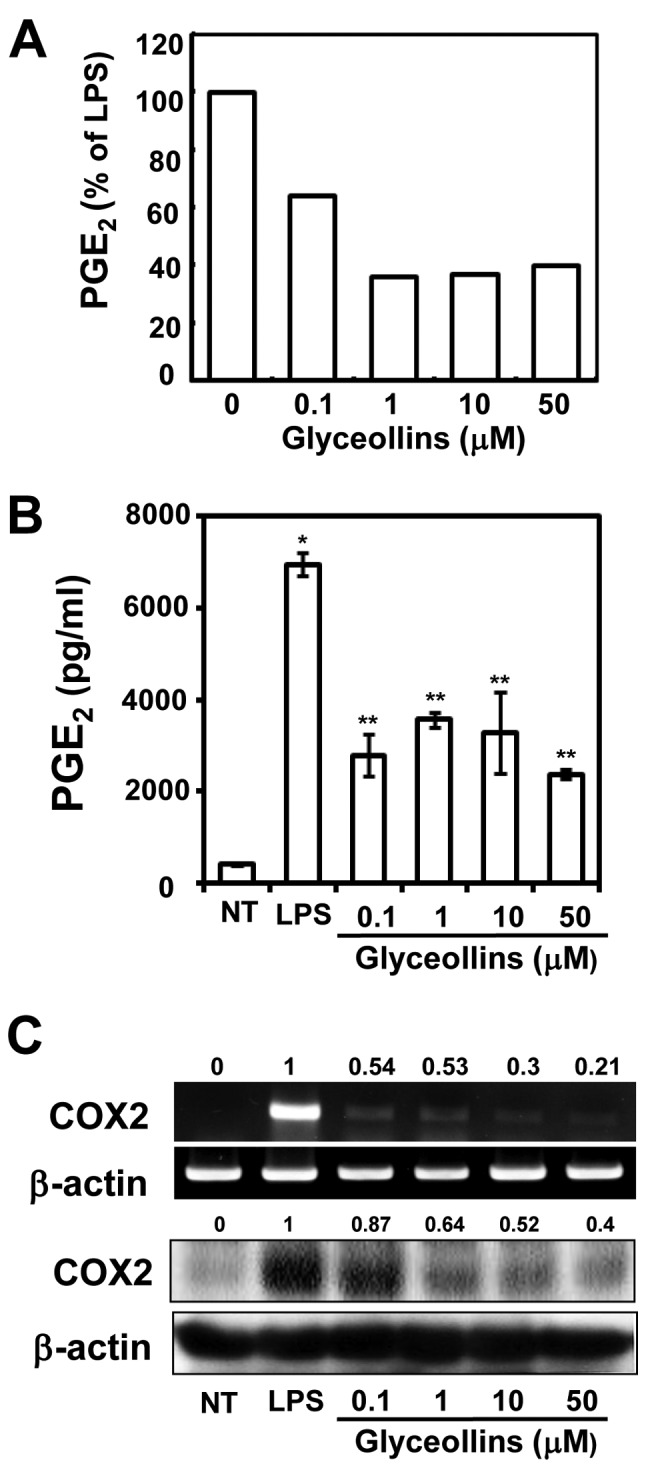
Effect of glyceollins on LPS-induced PGE_2_ production and COX-2 expression in RAW 264.7 cells. (A and B) RAW 264.7 cells were cultured in 6-well plates and were pre-incubated for 2 h with indicated concentrations of glyceollins before LPS (1 μg/ml) stimulation for 16 h. PGE_2_ amounts in cell supernatants were analyzed using an ELISA kit. Values are the means ± SD of three independent experiments. ^*^P<0.05, compared to the control; ^**^P<0.05, compared to the LPS treatment. (C) COX-2 mRNA and protein expression were examined by RT-PCR analysis with COX-2 primers or western blotting using an anti-COX-2 antibody.

To examine whether glyceollins inhibit PGE_2_ production, the cells were pre-incubated with glyceollins for 2 h and then activated with 1 μg/ml of LPS for 16 h. As shown in Fig. 2A, unstimulated RAW 264.7 cells mildly decreased PGE_2_ production when compared with treatment with glyceollins alone. In Fig. 2B, LPS induced a 5.2-fold increase in the biosynthesis of PGE_2_ as compared with untreated cells, but glyceollins strongly inhibited the production of PGE_2_ in a dose-dependent manner. Because the biosynthesis of PGE_2_ is catalyzed by COX-1 and COX-2 enzymes, we next measured the effect of glyceollins on LPS-induced COX-2 activities. We also detected that glyceollins inhibited the COX-2 mRNA and protein expressions in a dose-dependent manner (Fig. 2C, 5- and 2.5-fold, respectively). The data suggest that glyceollins can downregulate LPS-induced COX-2 expression at the transcription level. Inhibition of COX-2 expression by glyceollins was responsible for the decrease of PGE_2_ production.

### Effects of glyceollins on LPS-induced TNF-α, IL-1β, IL-18 and IL-10 mRNA expression

**Figure 3:**
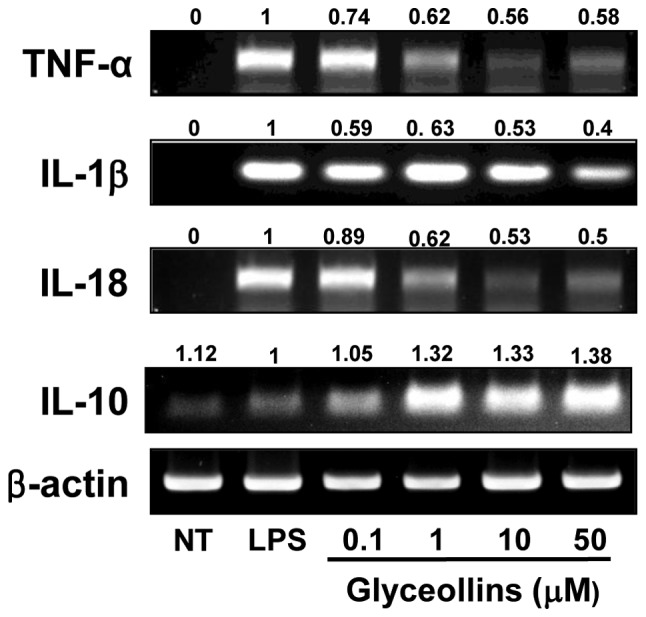
Inhibition of TNF-α, IL-1β and IL-18, and enhancement of IL-10 by glyceollins in RAW 264.7 cells. RAW 264.7 cells were pre-incubated with indicated concentrations of glyceollins for 2 h before LPS (1 μg/ml) stimulation for 16 h. Cells were harvested for RNA preparation. Transcriptional levels of cytokines were detected using RT-PCR. The figure shows a representative of three independent experiments.

Glyceollins were found to inhibit the pro-inflammatory mediators, such as NO and PGE_2_, most potently. Therefore, to test whether glyceollins could effectively for regulate inflammatory and anti-inflammatory cytokines in RAW 264.7 cells, we examined the mRNA levels of TNF-α, IL-1β, IL-18 and IL-10 by RT-PCR. Though IL-1β mRNA expression was only slightly inhibited in a dose-dependent manner (Fig. 3), TNF-α and IL-18 mRNA expression was significantly reduced by pretreatment of RAW 264.7 cells with glyceollins (0.1, 1, 10 or 50 μM) (Fig. 3). In contrast, in terms of the anti-inflammatory cytokines, the ability of glyceollins to induce IL-10 increased up to 38±0.5% in the LPS-activated RAW 264.7 cells (Fig. 3), while that of β-actin was not altered.

### Inhibitory effects of glyceollins on IκBα kinase activation and IκBα phosphorylation

**Figure 4:**
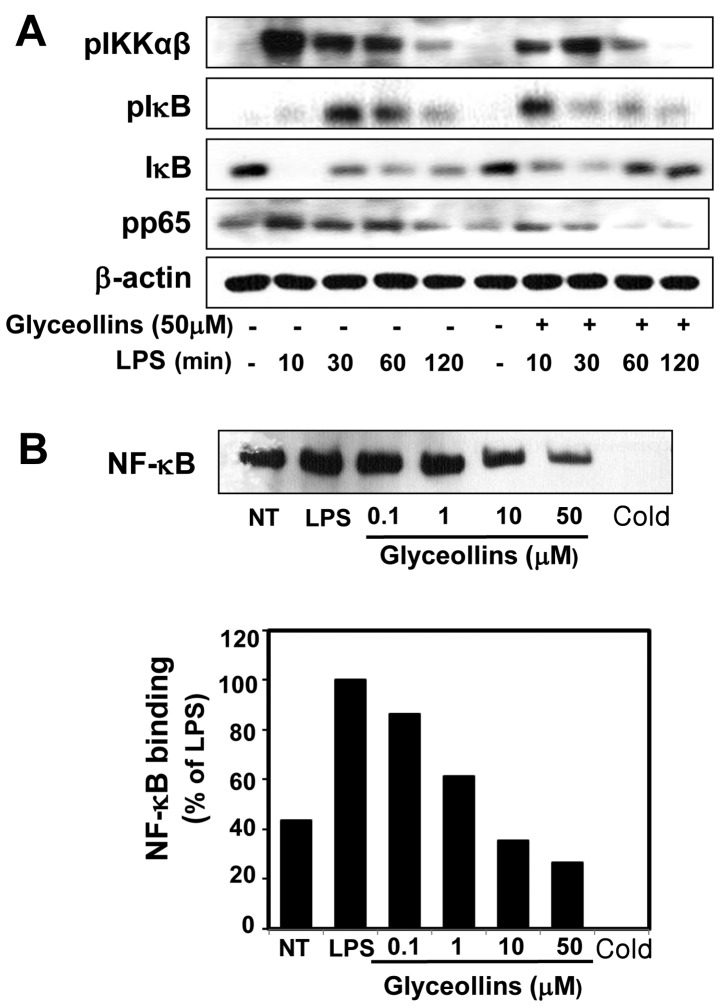
Inhibition of LPS-induced NF-κB activation by glyceollins. (A) Glyceollins blocking LPS-induced IκBα kinase activation and IκBα degradation. RAW 264.7 cells were pretreated with glyceollins at the indicated dose for 2 h and then stimulated with LPS (1 μg/ml) for 10, 30, 60 and 120 min. The protein extract was separated by SDS-PAGE followed by western blot analysis that was performed with specific antibodies. (B) Effect of glyceollins on LPS-induced NF-κB-DNA binding activity. RAW 264.7 cells were pretreated with glyceollins at the indicated doses for 2 h, and then stimulated with LPS (1 μg/ml) for 16 h. Nuclear extracts were prepared and EMSA was performed using a biotin-labeled NF-κB consensus binding sequence. Competitive EMSA using an unlabeled NF-κB consensus sequence at 100-fold excess confirmed the specificity of NF-κB protein binding. Three independent gels showed similar patterns.

Because the phosphorylation of IKK, and its subsequent phosphorylation of IκBα, are key signals for the activation of NF-κB, we examined the effect of glyceollins on LPS-induced phosphorylation of IKK and the degradation of IκBα protein by western blot analysis. A time-course experiment showed that IκBα in the cytoplasm was almost completely degraded within 10 min, and that it recovered at 30 min, after LPS (1 μg/ml) stimulation (Fig. 4A). Pretreatment with glyceollins prevented the induced degradation of IκBα protein at 5 and 15 min; the recovery of IκBα, which is under the control of NF-κB, was also suppressed (data not shown). IκBα phosphorylation was also examined by western blot analysis. As shown in Fig. 4A, the phosphorylated form of IκBα was hardly detectable in the resting RAW 264.7 cells; however, upon exposure to LPS (1 μg/ml) alone for 10 min, IκBα phosphorylation was initiated. At 30 min after LPS stimulation, pretreatment of glyceollins moderately inhibited LPS-mediated IκBα phosphorylation (Fig. 4A). Because IKK-α and β are the upstream kinases of IκB in the NF-κB signaling pathway and are activated via phosphorylation, we examined the effect of glyceollins on LPS-induced IKKα/β activations by western blot analysis using an a phospho-specific IKKα/β antibody. RAW 264.7 cells were pretreated with glyceollins (50 μM) for 2 h and then stimulated with LPS (1 μg/ml) for various courses of time. As shown in Fig. 4A, LPS was found to strongly induce IKKα/β phosphorylation, whereas glyceollin pretreatment significantly inhibited this phosphorylation. These findings indicate that glyceollins suppressed IKK and IκBα phosphorylation in LPS-induced RAW 264.7 cells.

### Effects of glyceollins on LPS-induced NF-κB activation

To investigate whether glyceollins affect the DNA binding ability of the NF-κB complex in the RAW 264.7 cells, we performed an electrophoretic mobility shift assay (EMSA). RAW 264.7 cells were pretreated with 0.1–50 μM of glyceollins for 2 h, and then the cells were stimulated with LPS for 16 h. RAW 264.7 cells with LPS strongly induced the DNA binding activity of NF-κB. In contrast, pretreatment with glyceollins significantly suppressed the induced the DNA-binding activity of NF-κB by LPS in a dose-dependent manner (Fig. 4B). Taken together, the above findings indicate that glyceollins suppress NO production as well as expressions of iNOS, COX-2, TNF-α, IL-1β and IL-18, at least in part via an NF-κB-dependent mechanism.

## Discussion

Phytoalexin is now a well-documented self-defense biomaterial that is produced by plants, and plays a critical role in exhibiting various biological events in animal and plant tissues (7,8). Recently, it has been revealed that these compounds have potential in ameliorating antioxidant, antifungal and antidiabetic activities *in vitro* and *in vivo* (34). In soybeans, glyceollin, a family of lipophilic phytoalexins, as a secondary metabolite, is often accumulated at sites infected by pathogens like *Phytophthora sojae* to inhibit their growth (35). The compound is also known to be induced by countless stress factors or physical stimuli, such as freezing, UV light exposure, and/or microbes (34,36). Because bean, grape, and sunflower seeds have been known to possess various biological activities, many researchers have been focused on the isolation and purification of active compounds; but this process was not convenient to obtain enough material for experimentation. The present approach of obtaining glyceollins deserves further study, especially since the connection between the signaling pathways and glyceollins has not been discovered so far. Although glyceollin and/or their derivatives have been investigated in relation to their biological activities, including anti-estrogenic activity (9–13), their precise mechanisms of signaling are scarcely understood at the present time.

Therefore, in the present study we first hypothesized that glyceollins effectively protect against the generation of pro-inflammatory mediators, specifically, NO and PGE_2_, through the inhibition of iNOS and COX-2 expression levels, respectively. We subsequently confirmed that the inhibition of NO production is concurrent with the suppression of iNOS expression at the mRNA and protein levels as shown by RT-PCR and western blot analysis (Fig. 1D). In addition, we investigated another mediator of inflammation, COX-2, which acts as a rate-limiting enzyme in the synthesis of prostaglandin-like PGE_2_. In line with our prediction, glyceollins strongly inhibited PGE_2_ production and COX-2 mRNA and protein levels (Fig. 2). TNF-α plays a key role in the induction of various genes, such as COX-2, through the activation of NF-κB by T cells and macrophages (37). To alleviate the inflammatory response in LPS-stimulated macrophages, it is also necessary to hamper IL-1β and IL-18 production, which both enhance the body's inflammatory response. These cytokines are known to be increased through the NF-κB signaling pathway (38–40). On the other side, IL-10 is a representative anti-inflammatory cytokine and has pleiotropic effects during the immunoregulation and inflammation processes in various immune cells (41). As shown in our data, glyceollins are effective for inflammation-related cytokines such as TNF-α, IL-1β, IL-18 or IL-10 expression using RT-PCR (Fig. 3). We clearly found that glyceollins are potent inhibitors of TNF-α production by RAW 264.7 macrophages with ~10 μM of an IC_50_ value; this is similar to luteolin and quercetin, which have been the most potent natural products so far at inhibiting TNF-α release, with IC_50_ values of <1 μM (42) and <5 μM, respectively (data not published).

It is well-recognized that NF-κB, a universal transcription factor, plays a pivotal role in the various soluble pro-inflammatory gene expressions and leukocyte adhesion molecules (38,39). As a key step to activate NF-κB functions, studies have been focused on the activation of the IKK complex over the last several years. Many natural phytochemicals, such as luteolin, quercetin, and resveratrol, suppress LPS-induced iNOS and COX-2 expressions as well as inflammatory cytokine expressions in macrophages by inhibiting the NF-κB signaling pathway, such as phosphorylation of IKK, degradation of IκBα, and nuclear translocation of NF-κB (43,44). Because the glyceollin-mediated signaling pathway can provide us with useful information on the function of the agent, we next studied the NF-κB-mediated signaling pathway in RAW 264.7 cells. In our study, western blot analysis revealed that glyceollins inhibited the LPS-induced phosphorylation of IKK, a series of phosphorylations of IκBα and degradation of p-IκB (Fig. 4A). We also demonstrated that glyceollins inhibit LPS-induced NF-κB in RAW 264.7 cells by performing EMSA. Pretreatment with glyceollins significantly suppressed the induced DNA-binding activity of NF-κB by LPS in a dose-dependent manner (Fig. 4B). Therefore, the above results suggest that glyceollins suppress the inflammation reaction through an NF-κB-dependent signal transduction pathway. Up to now, no data regarding a glyceollin-mediated signaling pathway has been investigated. As a result, our data have the potential to help develop prophylactic and therapeutic anti-inflammatory agents that are both safe and original. Further study of the overall signal transduction pathway promises to be rewarding because the inhibitory mechanism by glyceollins can provide new data for anti-inflammatory therapies.

This study describes a signaling pathway of the anti-inflammatory effects elicited glyceollins. Our results suggest that the glyceollins obtained from soybeans may exhibit increased anti-inflammatory effects. Glyceollins have an appreciable inhibitory activity against the overproduction of inflammatory mediators such as iNOS, COX-2 and various cytokines. Not only can they be used to investigate their effects in various biological areas, but if we uncover the precise mechanisms by glyceollins in immune cells, they can also be used to prevent immune diseases. Use of traditionally fermented soybean products may prove rewarding because we can naturally obtain their high levels of phytoalexins, such as glyceollins.

## Abbreviations

NO: nitric oxide
iNOS: inducible nitric oxide synthase
COX-2: cyclooxygenase-2
PGE_2_: prostaglandin
IKK: IκBα kinase
TNF: tumor necrosis factor
IL: interleukin

## References

[1] Torres N Torre-Villalvazo I Tovar AR Regulation of lipid metabolism by soy protein and its implication in diseases mediated by lipid disorders J Nutr Biochem 17 365 373 2006 16481155 10.1016/j.jnutbio.2005.11.005

[2] Ng TB Ye XJ Wong JH Fang EF Chan YS Pan W Ye XY Sze SC Zhang KY Liu F Wang HX Glyceollin, a soybean phytoalexin with medicinal properties Appl Microbiol Biotechnol 58 9059 9068 2011 10.1007/s00253-011-3169-7 21336922

[3] Song WO Chun OK Hwang I Shin HS Kim BG Kim KS Lee SY Shin D Lee SG Soy isoflavones as safe functional ingredients J Med Food 10 571 580 2007 18158825 10.1089/jmf.2006.0620

[4] Allred CD Allred KF Ju YH Geoppinger TS Doerge DR Helferich WG Soy processing influences growth of estrogen dependent breast cancer tumors Carcinogenesis 25 1649 1657 2005 10.1093/carcin/bgh178 15131010

[5] Messina M Soy foods and soybean phytoestrogens (isoflavones) as possible alternatives to hormone replacement therapy (HRT) Eur J Cancer 36 71 77 2000 10.1016/s0959-8049(00)00233-1 11056326

[6] Lee DS Lee SH Genistein, a soy isoflavone, is a potent alpha-glucosidase inhibitor FEBS Lett 501 84 86 2001 11457461 10.1016/s0014-5793(01)02631-x

[7] Jeandet P Douillet-Breuil AC Bessis R Debord S Sbaghi M Adrian M Phytoalexins from the Vitaceae: biosynthesis, phytoalexin gene expression in transgenic plants, antifungal activity, and metabolism J Agric Food Chem 50 2731 2741 2002 11982391 10.1021/jf011429s

[8] Boué SM Carter CH Ehrlich KC Cleveland TE Induction of the soybean phytoalexins coumestrol and glyceollin by *Aspergillus* J Agric Food Chem 82 167 172 2000 10.1021/jf9912809 10888516

[9] Burow ME Boué SM Collins-Burow BM Melnik LI Duong BN Carter-Wientjes CH Li S Wiese TE Cleveland TE McLachlan JA Phytochemical glyceollins, isolated from soy, mediate antihormonal effects through estrogen receptor α and β J Clin Endocrinol Metab 86 1750 1758 2001 11297613 10.1210/jcem.86.4.7430

[10] Nikov GN Hopkins NE Boue S Alworth WL Interactions of dietary estrogens with human estrogen receptors and the effect on estrogen receptor-estrogen response element complex formation Environ Health Perspect 108 867 872 2000 11017892 10.1289/ehp.00108867 PMC2556928

[11] Kim HJ Suh HJ Kim JH Park S Joo YC Kim JS Antioxidant activity of glyceollins derived from soybean elicited with *Aspergillus sojae* J Agric Food Chem 58 11633 11638 2010 21033668 10.1021/jf102829z

[12] Salvo VA Boué SM Fonseca JP Elliott S Corbitt C Collins-Burow BM Curiel TJ Srivastav SK Shih BY Wientjes CC Antiestrogenic glyceollins suppress human breast and ovarian carcinoma tumorigenesis Clin Cancer Res 12 7159 7164 2006 17145841 10.1158/1078-0432.CCR-06-1426

[13] Wood CE Clarkson TB Appt SE Franke AA Boué SM Burow ME McCoy T Cline JM Effects of soybean glyceollins and estradiol in postmenopausal female monkeys Nutr Cancer 56 74 81 2006 17176220 10.1207/s15327914nc5601_10

[14] Henkel T Zabel U Fanning E Baeuerle PA Intramolecular masking of the nuclear location signal and dimerization domain in the precursor for the p50 NF-κB subunit Cell 68 1121 1133 1992 1547506 10.1016/0092-8674(92)90083-o

[15] Karin M Delhase M The IκB kinase (IKK) and NF-κB: key elements of proinflammatory signaling Semin Immunol 12 85 98 2000 10723801 10.1006/smim.2000.0210

[16] Ghosh S Karin M Missing pieces in the NF-κB puzzle Cell 109 Suppl S81 S96 2002 11983155 10.1016/s0092-8674(02)00703-1

[17] Hayden MS Ghosh S Signaling to NF-κB Genes Develop 18 2195 2224 2004 15371334 10.1101/gad.1228704

[18] Zandi E Karin M Bridging the gap composition, regulation and physiological function of the IκB kinase complex Mol Cell Biol 19 4547 4551 1999 10373503 10.1128/mcb.19.7.4547 PMC84252

[19] Wang T Zhang X Li JJ The role of NF-κB in the regulation of cell stress responses Int Immunopharmacol 2 1509 1520 2002 12433052 10.1016/s1567-5769(02)00058-9

[20] Yang F Tang E Guan K Wang CY IKK plays an essential role in the phosphorylation of RelA/p65 on serine 536 induced by lipopolysaccharide J Immunol 170 5630 5635 2003 12759443 10.4049/jimmunol.170.11.5630

[21] Pahl HL Activators and target genes of Rel/NF-κB transcription factors Oncogene 18 6853 6866 1999 10602461 10.1038/sj.onc.1203239

[22] Wu JT Kral JG The NF-κB/IκB signaling system: a molecular target in breast cancer therapy J Surg Res 123 158 169 2005 15652965 10.1016/j.jss.2004.06.006

[23] Lang R Rutschman RL Greaves DR Murray PJ Autocrine deactivation of macrophages in transgenic mice constitutively overexpressing IL-10 under control of the human CD68 promoter J Immunol 168 3402 3411 2002 11907098 10.4049/jimmunol.168.7.3402

[24] Cheshire LL Baldwin AS Jr Synergistic activation of NF-κB by tumor necrosis factor α and interferon via enhanced IκBα degradation and de novo IκBα degradation Mol Cell Biol 17 6746 6754 1997 9343439 10.1128/mcb.17.11.6746 PMC232529

[25] Klostergaard J A rapid extremely sensitive, quantitative microassay for cytotoxic cytokines Lymphokine Res 4 309 317 1985 3876491

[26] Lowenstein CJ Alley EW Raval P Snowman AM Snyder SH Russell SW Murphy WJ Macrophage nitric oxide synthase gene: two upstream regions mediate induction by interferon gamma and lipopolysaccharide Proc Natl Acad Sci USA 90 9730 9734 1993 7692452 10.1073/pnas.90.20.9730 PMC47644

[27] Park CH Nam DY Son HU Lee SR Lee HJ Heo JC Cha TY Baek JH Lee SH Polymer fraction of *Aloe vera* exhibits a protective activity on ethanol-induced gastric lesions Int J Mol Med 27 511 518 2011 21286662 10.3892/ijmm.2011.609

[28] Pan MH Chang YH Tsai ML Lai CS Ho SY Badmaev V Ho CT Pterostilbene suppressed lipopolysaccharide-induced up-expression of iNOS and COX-2 in murine macrophages J Agric Food Chem 56 7502 7509 2008 18656926 10.1021/jf800820y

[29] Kusunoki N Kitahara K Kojima F Tanaka N Kaneko K Endo H Suguro T Kawai S Adiponectin stimulates prostaglandin E(2) production in rheumatoid arthritis synovial fibroblasts Arthritis Rheum 62 1641 1649 2010 20222108 10.1002/art.27450

[30] Ahn KS Noh EJ Zhao HL Jung SH Kang SS Kim YS Inhibition of inducible nitric oxide synthase and cyclooxygenase II by *Platycodon grandiflorum* saponins via suppression of nuclear factor-κB activation in RAW 264.7 cells Life Sci 76 2315 2328 2005 15748625 10.1016/j.lfs.2004.10.042

[31] Bradford MM A rapid and sensitive method for the quantitation of microgram quantities of protein utilizing the principle of protein-dye binding Anal Biochem 72 248 254 1976 942051 10.1016/0003-2697(76)90527-3

[32] Andújar I Recio MC Bacelli T Giner RM Ríos JL Shikonin reduces oedema induced by phorbol ester by interfering with IκBα degradation thus inhibiting translocation of NF-κB to the nucleus Br J Pharmacol 160 376 388 2010 20423347 10.1111/j.1476-5381.2010.00696.x PMC2874859

[33] Son M Kim A Lee J Park CH Heo JC Lee HJ Lee SH Ethanol extract of *Lycoris radiata* induces cell death in B16F10 melanoma via p38-mediated AP-1 activation Oncol Rep 24 473 478 2010 20596635 10.3892/or_00000881

[34] Paxton JD Biosynthesis and accumulation of legume phytoalexins Mycotoxins and Phytoalexins Sharma RP Salunkhe DK CRC Press Boca Raton 485 499 1991

[35] Rivera-Varga LI Schmitthenner AF Graham TL Soybean flavonoid effects on metabolism by *Phytophthora sojae* Phytochemistry 32 851 857 1993

[36] Feng S Saw CL Lee YK Huang D Fungal-stressed germination of black soybeans leads to generation of oxooctadecadienoic acids in addition to glyceollins J Agric Food Chem 55 8589 8595 2007 17892258 10.1021/jf0716735

[37] Andreakos E Targeting cytokines in autoimmunity: new approaches, new promise Expert Opin Biol Ther 3 435 447 2003 12783612 10.1517/14712598.3.3.435

[38] Chen F Demers LM Shi X Upstream signal transduction of NF-κB activation Curr Drug Targets Inflamm Allergy 1 137 149 2002 14561196 10.2174/1568010023344706

[39] Kulms D Schwarz T NF-κB and cytokines Vitam Horm 74 283 300 2006 17027519 10.1016/S0083-6729(06)74011-0

[40] Suk K Kim S Kim U Regulation of IL-18 production by IFN gamma and PGE_2_ in mouse microglial cells: involvement of NF-κB pathway in the regulatory processes Immunol Lett 77 79 85 2001 11377701 10.1016/s0165-2478(01)00209-7

[41] Mocellin S Panelli MC Wang E Nagorsen D Marincola FM The dual role of IL-10 Trends Immunol 24 36 43 2003 12495723 10.1016/s1471-4906(02)00009-1

[42] Paul AT Gohil VM Bhutani KK Modulating TNF-α signaling with natural products Drug Discov Today 11 725 732 2006 16846800 10.1016/j.drudis.2006.06.002

[43] Minich DM Bland JS Dietary management of the metabolic syndrome beyond macronutrients Nutr Rev 66 429 444 2008 18667004 10.1111/j.1753-4887.2008.00075.x

[44] Ruiz PA Haller D Functional diversity of flavonoids in the inhibition of the proinflammatory NF-kappaB, IRF, and Akt signaling pathways in murine intestinal epithelial cells J Nutr 136 664 671 2006 16484540 10.1093/jn/136.3.664

